# Comprehensive rehabilitation with integrative medicine for subacute stroke: A multicenter randomized controlled trial

**DOI:** 10.1038/srep25850

**Published:** 2016-05-13

**Authors:** Jianqiao Fang, Lifang Chen, Ruijie Ma, Crystal Lynn Keeler, Laihua Shen, Yehua Bao, Shouyu Xu

**Affiliations:** 1 Department of Acupuncture, The Third Affiliated Hospital of Zhejiang Chinese Medical University, 219 Moganshan Road, Xihu District, Hangzhou City, Zhejiang Province 310005, China; 2 Zhejiang Chinese Medical University, 548 Binwen Road, Binjiang District, Hangzhou City, Zhejiang Province 310053, China; 3 Department of Acupuncture & Encephalopathy, Jiaxing Hospital of Traditional Chinese Medicine, 1501 Zhong shan East Road, Jiaxing City, Zhejiang Province 310012, China; 4 Department of Acupuncture & Rehabilitation, Hangzhou Hospital of Traditional Chinese Medicine, 453 Tiyuchang Road, Xihu District, Hangzhou City, Zhejiang Province 310007, China; 5 Department of Rehabilitation, The Third Affiliated Hospital of Zhejiang Chinese Medical University, 219 Moganshan Road, Xihu District, Hangzhou City, Zhejiang Province 310005, China

## Abstract

To determine whether integrative medicine rehabilitation (IMR) that combines conventional rehabilitation (CR) with acupuncture and Chinese herbal medicine has better effects for subacute stroke than CR alone, we conducted a multicenter randomized controlled trial that involved three hospitals in China. Three hundred sixty patients with subacute stroke were randomized into IMR and CR groups. The primary outcome was the Modified Barthel Index (MBI). The secondary outcomes were the National Institutes of Health Stroke Scale (NIHSS), the Fugl-Meyer Assessment (FMA), the mini-mental state examination (MMSE), the Montreal Cognitive Assessment (MoCA), Hamilton’s Depression Scale (HAMD), and the Self-Rating Depression Scale (SDS). All variables were evaluated at week 0 (baseline), week 4 (half-way of intervention), week 8 (after treatment) and week 20 (follow-up). In comparison with the CR group, the IMR group had significantly better improvements (*P* < 0.01 or *P* < 0.05) in all the primary and secondary outcomes. There were also significantly better changes from baseline in theses outcomes in the IMR group than in the CR group (*P* < 0.01). A low incidence of adverse events with mild symptoms was observed in the IMR group. We conclude that conventional rehabilitation combined with integrative medicine is safe and more effective for subacute stroke rehabilitation.

There are 1.5–2 million new strokes in China each year, and the burden of stroke is increasing rapidly^1^. Stroke treatment has developed rapidly in China since the early 1980s, and stroke units are still evolving to adopt a more standardized form of care^2^. Integrative medicine that combines complementary therapies (like traditional Chinese medicine) and conventional allopathic interventions is becoming more common and widely accepted across the world. As integrative medicine develops, more and more stroke units in China have utilized traditional Chinese medicine (TCM) to adapt to the demands of an increasing patient population. China has begun to establish more standardized stroke treatment modalities that include a comprehensive approach^3^. This comprehensive strategy, named integrative medicine rehabilitation (IMR), incorporates traditional Chinese therapies, such as acupuncture and Chinese herbal medicine along with conventional rehabilitation (CR).

Previous studies showed that acupuncture can reduce injury volume and exert a neuroprotective role in experimental ischemic stroke^4^,^5^. A systematic review also suggested that acupuncture may be effective in the treatment of post-stroke rehabilitation without significant side effects^6^. Chinese herbal medication has also been demonstrated to have neuroprotective effects on cerebral ischemia^7^,^8^. However, most of the published studies only investigated the effects of acupuncture or Chinese medications alone, whether a combination of these treatments has better effect on stroke recovery is still unknown^9^.

We conducted a prospective, two-group, randomized, controlled, assessor-blinded trial across three centers. In this trial, we designed prescriptions of Chinese herbal medicine and acupuncture for subacute stroke according to four common types of pattern differentiation recommended as a standard treatment for stroke in China. With a holistic view of the human body, we conducted a combined treatment of acupuncture and Chinese herbal medicine to regulate the overall and delicate harmony and balance of the body. This study aimed to determine whether integrative medicine rehabilitation (which combines conventional rehabilitation with the complex treatment of acupuncture and Chinese herbal medication based on syndrome differentiation) is more effective than conventional rehabilitation in improving activities of daily living (ADL) and neurologic deficits of stroke (motor dysfunction, cognitive impairment, and post-stroke depression) during the subacute stage.

## Results

### Study Participants

We recruited participants from two large cities (Hangzhou and Jiaxing, China) via advertisements by local newspapers, health-related TV programs, Internet, and posters in communities and hospitals between March 1, 2012 and December 25, 2014. The clinical trial ended on May 30, 2015 when we completed follow-up of the last subject.

**Figure 1:**
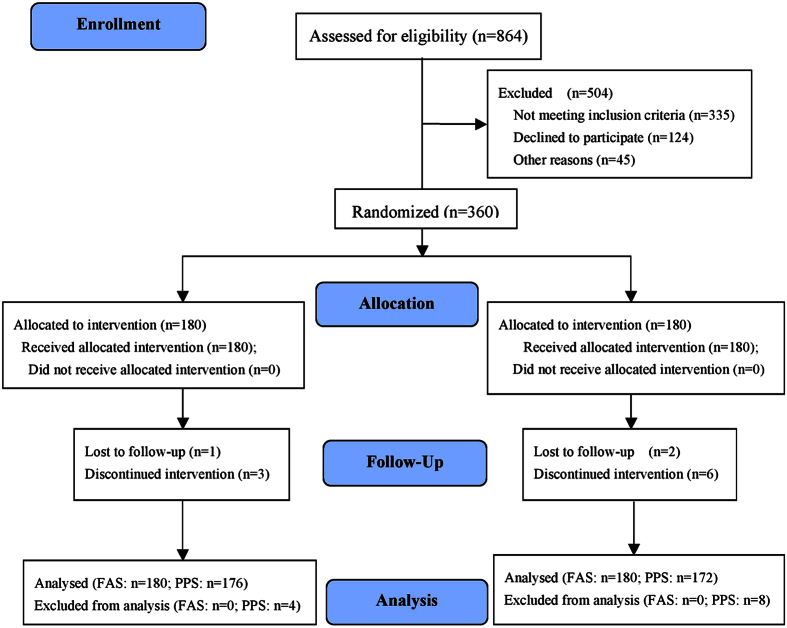
Flow of participants through the trial.

Approximately 864 evaluations yielded the desired sample size of 360 randomized participants (Fig. 1). Of the 360 subjects, 131 (36.39%) passed a screening for cognitive impairment and 153 (42.50%) passed a screening for post-stroke depression (PSD). Of the twelve subjects who dropped out, three were lost to follow-up in two groups; two failed to complete treatment, and one in the IMR group suffered from aspiration pneumonia. Two subjects withdrew because of recurrent stroke and four subjects in the CR group were excluded because of their violations of the treatment restriction (receiving additional TCM treatment). In total, 176 participants of the IMR group and 172 participants of the CR group completed all of the treatments and measurements, and were included in the per-protocol set (PPS), which indicated very good compliance and tolerability of our intervention. No participants died during the study period. A total of 180 subjects of each group were included in the full-analysis set (FAS). The efficacy results of the PPS and FAS were consistent, so we presented efficacy results here only from the PPS. The two groups did not differ in pretreatment demographic and clinical variables at baseline, as shown in Table 1.

### Primary and Secondary Outcomes

**Figure 2:**
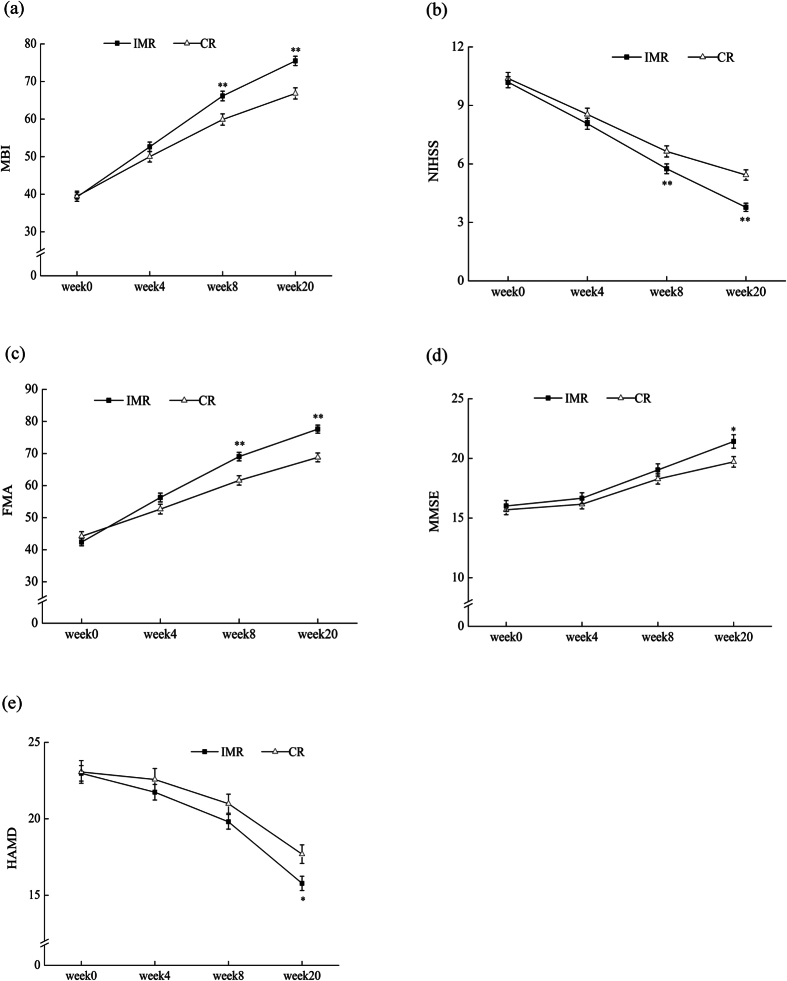
Means of MBI, NIHSS, FMA, MMSE, HAMD at Four Testing Time points. (**a**) MBI score for the IMR and CR at four testing time points (mean ± SEM; IMR n = 176, CR n = 172). **P < 0.01, compared to CR group. (**b**) NIHSS score for the IMR and CR at four time points (mean ± SEM; IMR n = 176, CR n = 172). **P < 0.01, compared to CR group. (**c**) FMA score for the IMR and CR at four testing time points (mean ± SEM; IMR n = 176, CR n = 172). **P < 0.01, compared to CR group. (**d**) MMSE score for the IMR and CR at four testing time points (mean ± SEM; IMR n = 62, CR n = 69). *P < 0.05, compared to CR group. (**e**) HAMD score means for the IMR and CR at four testing time points (mean ± SEM; IMR n = 76, CR n = 77). *P < 0.05, compared to CR group.

All primary and secondary variables at different stages are showed in Supplemental Table S1. The Modified Barthel Index (MBI) is a reliable measure of functional independence with a total score of 100^10^,^11^. We used this index as a primary outcome to evaluate activities of daily life. Patients in the IMR group exhibited a significant improvement in MBI (Interaction effect: *F* (3, 344) = 37.70, *P* < 0.001, Repeated-measures ANOVA) (Table 2) and a better improvement from baseline than patients in the CR group (36.25 ± 13.05 vs. 27.61 ± 13.72, 95% CI: 5.86 ∼ 11.41, *T* = 6.12, *P* < 0.001, two-tailed unpaired Student’s *t*-test) (Table 3). Significant differences between the two groups were observed at week 8 (66.04 ± 17.09 vs. 59.56 ± 19.62, *F* = 11.17, *P* = 0.001, multivariate ANOVA) and week 20 (75.36 ± 16.64 vs. 66.64 ± 19.60, *F* = 20.72, *P* < 0.001) (Fig. 2a).

For neurological deficits, the National Institutes of Health Stroke Scale (NIHSS) strongly predicts the likelihood of a patient’s recovery after stroke. A low score indicates a good neurological function^12^. Both the means of NIHHS (Interaction effect: *F* (3, 344) = 9.08, *P* < 0.001) and the changes from baseline (−6.37 ± 2.66 vs. −4.94 ± 2.76, 95% CI: −1.99 ∼−0.87, *T* = −4.99, *P* < 0.001) were significantly different (Tables 2 and 3). Figure 2b indicates the difference between the two groups, with the IMR group having significantly lower scores at week 8 (5.63 ± 3.39 vs. 6.87 ± 4.08, *F* = 9.89, *P* = 0.002) and week 20 (3.69 ± 2.85 vs. 5.67 ± 3.82, *F* = 30.99, *P* < 0.001).

The Fugl-Meyer Assessment (FMA), with a total possible score of 100 points, was included as another secondary outcome to assess motor function^13^. There was a significant improvement in FMA in the IMR group (Table 2, Interaction effect: *F* (3, 344) = 17.51, *P* < 0.001; Table 3, 33.58 ± 13.67 vs. 24.45 ± 13.01, 95% CI: 6.36 ∼ 11.89, *T* = 6.49, *P* < 0.001). Figure 2c illustrates higher mean values of FMA in the IMR group compared with the CR group at week 8 (69.01 ± 17.75 vs. 61.55 ± 19.19, *F* = 14.65, *P* < 0.001) and week 20 (77.44 ± 16.84 vs. 68.68 ± 18.03, *F* = 22.67, *P* < 0.001).

We used the adjusted scores of the mini-mental state examination (MMSE) and the Montreal Cognitive Assessment (MoCA) with new cutoffs (MMSE ≤ 24 and MoCA ≤ 20) as diagnostic criteria to maximally reduce the influence of age and education^14^. Approximately 62 participants in the IMR group and 69 in the CR group were eligible for cognitive impairment with comparable baseline (Table 1). There were significant differences between the two groups in mean values (Interaction effect: MMSE: *F* (3, 127) = 3.09, *P* = 0.029; MoCA: *F* (3, 127) = 8.55, *P* < 0.001) (Table 2), and in mean changes from baseline (MMSE: 5.40 ± 3.23 vs. 4.01 ± 2.64, 95% CI: 0.41 ∼ 2.37, *T* = 2.81, *P* = 0.006; MoCA: 5.32 ± 1.91 vs. 3.81 ± 1.87, 95% CI: 0.86 ∼ 2.16, *T* = 4.57, *P* < 0.001) (Table 3). Figure 2d shows that the mean MMSE score in the IMR group was higher at week 20 (21.42 ± 4.47 vs. 19.71 ± 3.69, *F* = 5.74, *P* = 0.018).

Post-stroke depression (PSD) is also a common symptom after stroke. A total of 76 cases in the IMR group and 77 cases in the CR group were diagnosed to have PSD according to a 24-item HAMD score ≥8^15^. The baselines of HAMD and SDS in the two groups were comparable (*P* = 0.919; *P* = 0.305) (Table 1). The means of both variables were significantly different between the two groups (Interaction effect: HAMD: *F* (3, 149) = 9.77, *P* < 0.001; SDS: *F* (3, 149) = 8.08, *P* = 0.029) (Table 2), and the changes from baseline were also significantly bigger in IMR compared with CR (HAMD: −7.20 ± 3.08 vs. −5.38 ± 4.37, 95% CI: −3.03∼−0.61, *T* = −2.98, *P* = 0.003; SDS: −16.31 ± 9.30 vs. −10.34 ± 8.73, 95% CI: −8.67∼−2.91, *T* = −3.97, *P* < 0.001) (Table 3). Figure 2e shows that there were significant differences in means with HAMD at week 20 (15.78 ± 4.09 vs. 17.69 ± 5.32, *F* = 6.20, *P* = 0.014).

### Safety Results

We summarized the adverse reactions to acupuncture and Chinese medication. Side effects such as bleeding, local hematoma, and prickling were inevitable for invasive therapy with acupuncture. Adverse reactions to Chinese medicines were monitored by lab tests (liver and kidney function tests and three routine tests of blood, urine and stool) and documentation of allergies and symptoms of gastrointestinal discomfort. Table 4 shows mild symptoms with low incidence and rare adverse events in the IMR group during the study period (Please see Supplemental Table S2. Adverse Events Reported in Study).

## Discussion

This trial demonstrated that integrative medicine rehabilitation had better effects than conventional rehabilitation alone in improving ADL, neurological impairment, motor function, cognitive impairment and PSD as long-term outcomes. The beneficial results on all outcome measurements during the follow-up period indicated that this comprehensive rehabilitation focused on the adjustment of overall function may contribute to many aspects of stroke recovery. The ultimate goal of stroke rehabilitation should always be focused on improving ADL of patients so that they could achieve greater independence^16^, which was the primary outcome measured by MBI and was considerably improved in this trial. The NIHSS is a neurologic severity scale developed mainly for use in acute-stroke therapy trials and early prediction of functional abilities^17^, but we found it sensitive in our subacute stroke trial. We used the FMA motor subscale to measure motor function of upper and lower extremities, and observed positive improvements. The results of multivariate ANOVA indicated that the mean MBI and FMA both significantly improved at week 8, which were earlier than improvement in cognitive function and PSD at week 20. These findings suggested that the improvement of limb function may greatly contribute to the recovery of ADL, which is consistent with the view that motor function is particularly important in carrying out ADL^18^.

Cognitive impairment and depression are two common sequelae after stroke that require early diagnosis and treatment. We used the MMSE, MoCA, HAMD, and SDS as assessment tools. The HAMD has been the “gold standard” in antidepressant clinical trials^19^. The SDS is a self-reported instrument that is usually used to screen for depression in clinical practice. We applied both scales to measure PSD and used the HAMD as a diagnostic item. In the MoCA test for evaluating cognitive impairment, many patients in our trial could not name a rhinoceros because they had never seen this animal. In contrast, the MMSE seemed more applicable in our trial, particularly for uneducated or older participants, which was consistent with previous studies^20^. The results of multivariate ANOVA for comparing means at each testing time point indicated that IMR improved cognitive function and depression at the same time point (week 20).

In terms of the safety of acupuncture and Chinese medication, a very low incidence of mild symptoms and low incidence of adverse events were observed in this trial. As long as acupuncture is performed by trained practitioners using the clean needle technique, it is usually a safe procedure^21^. Prescription of Chinese herbs under pattern identification principles, excluding the use of any toxic herbs, may provide maximum safety. In the hands of experienced practitioners, Chinese herbs are very safe^22^.

Pattern identification (or called syndrome differentiation and treatment) and holistic assessment and diagnosis are two key elements in traditional Chinese medicine. TCM “syndromes” reflect the pathogenesis and pathophysiology of a given case and provide a basis for prevention and treatment^23^,^24^. We designed the prescriptions of acupoints and Chinese herbs under the principles of these two key elements. The use and modification of acupoints and herbs in all subjects were all made according to the differentiation and treatment of their specific syndromes. The combined use of acupuncture and Chinese herbal medicine could more comprehensively regulate organs and meridians. Furthermore, they may overcome each other’s disadvantages.

Although there have been a large number of randomized controlled trials on acupuncture or Chinese herbal medicine over the past twenty years, most of them were dedicated to revealing the effects of a single modality or only for one particular dysfunction after stroke. Because acupuncture and Chinese herbal medicine may have beneficial effects on many different stroke sequelae, why not use their combination to treat these disorders simultaneously? In reality, patients with stroke not only suffer from motor disorders but also experience PSD, cognitive impairment, or other dysfunctions at the same time. Integrative medicine rehabilitation for stroke patients may have beneficial effects on all dysfunctions that are responsive to personalized treatment of acupuncture and Chinese herbs. In fact, this kind of integrative approach is consistent with common clinical practice in China. Treatment with acupuncture and Chinese medication with multiple goals may produce better and more comprehensive results. Our trial treated patients with hemiplegia, cognitive disorders and PSD simultaneously. The results provide strong evidence that comprehensive rehabilitation approach has multiple effects on ischemic stroke during the subacute stage.

There are several limitations that should be acknowledged. During the trial, one patient with dysphagia suffered aspiration pneumonia caused by taking the herbal decoction orally. We used decoctions (herbs boiled with water) because it is the most common method of applying herbal therapy and is generally regarded as fast and strong in action. However, not all of the patients with dysphagia received nasogastric tube feeding, and many of them were fed orally. To avoid the occurrence of this treatment-related adverse event, we were required to remove dysphagia from the protocol. Another limitation is that we did not use sham acupuncture or placebo herbal medicine in the control group. Without controlling placebo effect, the results of the study may include a potential placebo bias. Because many Chinese patients have previous experience with acupuncture and herbal medicine or know about them, it was impractical to blind them with sham treatments. Moreover, the literature on sham acupuncture is extremely heterogeneous in design and implementation, with highly variable methods^25^. This area of study is quite specialized and is out of the scope of this project. Instead, we paid close attention to reducing bias by minimizing interaction during treatments in the IMR group and communication among participants of the two groups.

## Conclusions

In conclusion, the method of integrative medicine rehabilitation in our study features the following two aspects: 1. The diagnosis, treatment and care, conventional rehabilitation, efficacy assessment and safety detection based on Western medicine; 2. The application of a unique way of diagnosing syndrome differentiation and treatment with acupuncture and Chinese herbal medicine. These combined techniques constitute a comprehensive rehabilitation strategy. Our trial demonstrated that treating subacute stroke with the combination of Western medicine and complex TCM treatments (including acupuncture and Chinese herbal medication) improved overall outcomes of stroke rehabilitation.

## Methods

### Study design and ethics

This 20-week clinical trial was conducted in accordance with the Declaration of Helsinki, registered at Chictr.org (number: ChiCTR-TRC-12001972; date: March 1, 2012), and reported according to CONSORT guidelines. The details of the trial protocol have been published previously^26^. This study was approved as appropriate by the ethics committees of the Third Affiliated Hospital of Zhejiang Traditional Chinese Medical University, Hangzhou Hospital of Traditional Chinese Medicine and Jiaxing Hospital of Traditional Chinese Medicine. We recruited participants by advertising in local newspapers, health-related TV programs, Internet, and posters in the above three hospitals and communities in Hangzhou city and Jiaxing city. All participants were assigned to treatment only after they had signed their informed consent.

### Participants

Patients aged 35–80 years old with a recent (30–40 days) ischemic stroke, an NIHSS score of 5–15, and a modified Rankin Scale (mRS) score of 2–5 were included. Patients who had the first incidence of stroke or patients who had a history of stroke but without disability (mRS ≤1) were potential candidates for inclusion.

The exclusion criteria eliminated patients with serious heart, liver, kidney, or hematopoietic system-related diseases, or serious psychiatric disorders. Any patients who had received thrombolytic therapy or TCM treatment, participated in other clinical trials in last three months, were pregnant or breast-feeding, or had been born with congenital disabilities were also excluded. Patients who could not receive acupuncture and Chinese medicine treatment were excluded from this study. At the beginning of the study, a patient with dysphagia suffered aspiration pneumonia after taking Chinese herbal decoction orally. To eliminate such treatment-related adverse events, patients with swallowing disorders, which was originally designed as one of the secondary outcomes, were excluded from our trial. We also excluded subjects with minor or severe stroke (the NIHSS score was between 4 and 24 in our original protocol) after the trial commencement, because of the more frequent and intensive care required in severe stroke patients that was beyond our treatment program, and the early discharge in minor stroke patients.

### Randomization and Blinding

Subjects were randomly assigned to receive either IMR or CR at a 1:1 ratio using an Excel generated random number list. The randomization sequence was placed into sequentially numbered, opaque, sealed envelopes, which were saved by special screeners. All of the rehabilitation therapists, outcome assessors, and data analysts were blinded to group assignments.

### Interventions and comparison

Both groups received conventional stroke rehabilitation treatment during the entire 20-week study period. To examine the impact of Chinese integrative medicine on the treatment group, the conventional rehabilitation therapy was similarly applied in both groups. All rehabilitation therapists were blinded to the group assignment of the participants. Rehabilitation treatments were conducted by certified therapists with more than five years of experience with stroke patients specifically, and were trained according to the investigator’s brochure. The program was designed according to the Chinese stroke rehabilitation treatment guidelines^27^. For motor disorder, Bobath techniques and traditional rehabilitation treatments (includes normal limb posture, ROM exercises, muscle strengthening exercises) were used. *Bobath concept: theory and clinical practice in neurological rehabilitation* describes the major techniques used^28^. Among the above therapies, the selection of specific treatment items and intensity varied according to patients’ individual conditions. Participants in both groups who met the criteria for cognitive impairment received an additional one-hour cognitive training conducted by a speech therapist. Participants in both groups who met the criteria for post-stroke depression received an additional one-hour psychological therapy conducted by a psychologist. The conventional rehabilitation program was performed five days per week for 20 weeks. All participants of both groups were recommended to be hospitalized to receive the 20-week free conventional rehabilitation treatment. Each subject had a therapy card that recorded his/her name, randomized number and rehabilitation program (included contents and time). The therapists signed their names after every treatment, but were blinded to the group information as well the assessors. The IMR group received 30 additional minutes of acupuncture therapy as a bedside treatment in a supine position once a day six days per week for eight weeks, and a Chinese herbal decoction twice a day for eight weeks.

#### Acupuncture treatment

The acupuncture program was performed by certified acupuncturists with more than five years of clinical experience using filiform steel needles (size 0.25 mm × 40 mm, Huatuo brand, manufactured by Suzhou Medical Appliance in Suzhou, Jiangsu Province China). The basic prescription of acupoints (including scalp acupuncture and body acupuncture) and modifications according to different sequelae and pattern identifications are detailed in Table 5. Electro-acupuncture was applied to LI15 (Jianyu), LI11 (Quchi), ST36 (Zusanli), and GB39 (Xuanzhong) points using GB6805-2 Electro-Acu Stimulators (Huayi Medical Supply & Equipment Co., Ltd, Shanghai, China). The parameter was a 2-Hz intermittent wave at an intensity within the patients’ tolerance. To minimize social interaction between the acupuncturists and subjects, needles were inserted in 5–7 minutes and the subjects were left alone to rest for 30 minutes, with no additional attention, training, or interaction. Needles were removed quickly within 3–5 minutes, and social interaction during the acupuncture session was minimal.

#### Chinese medication

The prescription of Chinese herbs was carried out by TCM doctors, which was based on the same four types of pattern identification as acupuncture (contents of Chinese herbal medicine detailed in Table 5). This “syndrome differentiation” for sub-acute stroke treatment is clearly outlined in *Traditional Chinese Medicine Diagnosis and Treatment Program including 95 Diseases of 22 Departments* (a government document published by the Bureau of Public Republic of Traditional Chinese Medicine, 2010: Beijing, Chinese version only).

### Outcome measures

The primary outcome was the MBI for ADL. The secondary outcomes were the NIHSS for neurological deficits, the FMA motor subscale for motor dysfunction of the upper and lower extremities, the MMSE and MoCA for cognitive impairment, and the HAMD and SDS for post-stroke depression. All outcomes were evaluated at four testing timepoints: week 0 (baseline), week 4 (midway of intervention period), week 8 (after treatment), and week 20 (follow-up). Safety and tolerability were assessed at each visit. Three changes were made regarding the registration platform in our final study protocol: swallowing disorder was excluded at the very beginning of the trial to avoid treatment-related aspiration pneumonia; MBI was added as the primary outcome to measure the daily living ability of patients toward greater independence; and the follow-up period was prolonged from sixty days to 12 weeks.

### Statistical analysis

Sample size calculations were based on our preliminary test and previous studies^29^ and detailed in our study protocol^26^. All analyses were conducted by a statistician blinded to group allocation at the Clinical Research Institute of Zhejiang Provincial Hospital of Traditional Chinese Medicine using the SPSS (Version17.0) statistical package. A P-value (two sided) of less than 0.05 was considered to represent statistical significance. The analysis of MBI, HINSS, FMA and safety were conducted in the full analysis set (FAS) according to the intention-to-treat (ITT) principle. The per protocol set (PPS) was also conducted in the sensitivity analysis for all of variables. The analysis of cognitive impairment and PSD was made among the defined population of corresponding dysfunction (Table 1, legend c). Missing values were handled by the mixed model for repeated measurements (MMRM). Continuous variables in this trial, which were all tested with normal distribution, were expressed as mean ± SD. Categorical variables were expressed as numbers (%). Repeated-measure ANOVA was used to compare differences in all variable means across time by group and time. Multivariate ANOVA was used to compare the means of the two groups at each testing time point. A two-tailed unpaired Student’s t-test was used to compare the differences in value changes (follow-up minus baseline) between the two groups. A χ2 test was used in the statistical analysis for categorical variables of entry.

## Additional Information

**How to cite this article**: Fang, J. *et al*. Comprehensive rehabilitation with integrative medicine for subacute stroke: A multicenter randomized controlled trial. *Sci. Rep.* **6**, 25850; doi: 10.1038/srep25850 (2016).

## Supplementary Material

Supplementary Information

## Figures and Tables

**Table 1:** Baseline Characteristics.

Characteristics	IMR (N = 180)	CR (N = 180)
Age, y, mean (SD)	64.5 (11.9)	65.8 (11.3)
Male, gender, n (%)	96 (53.6%)	111 (61.3%)
Education level (years)^a^		
0–5, n (%)	38 (21.11%)	34 (18.89%)
5–8, n (%)	64 (35.56%)	80 (44.44%)
>8, n (%)	78 (43.33%)	66 (36.67%)
History of stroke, mean (SD), d	34.09 (2.64)	34.47 (2.69)
Side of hemiparesis, n (%)		
Left	109 (60.56)	110 (61.11)
Right	71 (39.44)	70 (38.89)
Vascular risk factors		
Hypercholesterolemia, n (%)	94 (52.22)	100 (55.56)
Hypertension, n (%)	110 (61.11)	114 (63.33)
Diabetes mellitus, n (%)	56 (31.11)	52 (28.89)
Syndrome differentiation^b^		
Type 1	36 (20.00%)	49 (27.22%)
Type 2	43 (23.89%)	42 (23.33%)
Type 3	65 (36.11%)	62 (34.44%)
Type 4	36 (20.00%)	27 (15.00%)
MBI, mean (SD)	39.26 (15.50)	39.48 (17.61)
NIHSS, mean (SD)	10.19 (3.82)	10.38 (4.07)
FMA, mean (SD)	43.86 (16.07)	44.23 (18.52)
Cognitive impairment, n (%)	62 (34.44)	69 (38.33)
MMSE, mean (SD)^c^	16.02 (3.57)	15.70 (3.43)
MoCA, mean (SD)^c^	14.65 (3.10)	13.96 (3.14)
PSD, n (%)	76 (42.22)	77 (42.78)
HAMD, mean (SD)^c^	22.97 (4.42)	23.06 (6.05)
SDS, mean (SD)^c^	64.94 (5.40)	63.95 (6.32)

Abbreviations: IMR, Integrative medicine rehabilitation; CR, Conventional rehabilitation; MBI, Modified Barthel Index; NIHSS, The National Institutes of Health Stroke Scale; FMA, Fugl-Meyer Assessment; MMSE, The Mini-Mental State Examination; MoCA, Montreal Cognitive Assessment; PSD, post-stroke depression; HAMD, Hamilton’s Depression Scale; SDS, Self-Rating Depression Scale.

^a^Education level: 0–5 years means elementary school, 5–8 years means middle school, >8 years means above high middle school.

^b^Syndrome differentiation: Type 1, Disturbance of wind-fire; Type 2, Phlegm-stasis blocking collateral; Type 3, Yin deficiency and wind act; Type 4, Qi deficiency and blood stasis.

^c^The analysis of cognitive impairment (MoCA, MMSE) and post-stroke depression (HAMD, SDS) were made on the cases who tested with MMSE ≤24 & MoCA ≤20 and HAMD ≥8, respectively.

**Table 2:** Results of Repeated Measures ANOVA of all Variables (PPS).

Variables	*df*	*df*	Effects	*F*	*P*	Partial Eta Squared
	Hypothesis	Error				
MBI	1	346	Group	5.95	0.015	0.017
	3	344	Time	670.62	<0.001	0.854
	3	344	Group by time	37.70	<0.001	0.103
NIHSS	1	346	Group	8.90	0.003	0.011
	3	344	Time	542.29	<0.001	0.819
	3	344	Group by time	9.08	<0.001	0.115
FMA	1	346	Group	2.74	0.099	0.008
	3	344	Time	519.56	<0.001	0.819
	3	344	Group by time	17.51	<0.001	0.053
MMSE	1	129	Group	1.80	0.182	0.014
	3	127	Time	136.86	<0.001	0.764
	3	127	Group by time	3.09	0.029	0.068
MoCA	1	129	Group	5.28	0.023	0.039
	3	127	Time	296.01	<0.001	0.875
	3	127	Group by time	8.55	<0.001	0.168
HAMD	1	151	Group	1.61	0.207	0.011
	3	149	Time	139.68	<0.001	0.738
	3	149	Group by time	9.77	<0.001	0.102
SDS	1	151	Group	2.037	0.156	0.013
	3	149	Time	110.25	<0.001	0.689
	3	149	Group by time	8.08	0.029	0.104

**Table 3:** Value Changes from Baseline (week 20-week 0) of Variables Compared by Independent Samples *t*-test (PPS).

Variable	Group (N)	Week 20-week 0 Mean(SD)	95% CI of the Difference	*T*	*P*
MBI	IMR (176)	36.25 (13.05)	5.86, 11.41	6.12	<0.001
	CR (172)	27.61 (13.72)			
NIHSS	IMR (176)	−6.37 (2.66)	−1.99, −0.87	−4.99	<0.001
	CR (172)	−4.94 (2.76)			
FMA	IMR (176)	33.58 (13.67)	6.36, 11.89	6.49	<0.001
	CR (172)	24.45 (13.01)			
MMSE	IMR (62)	5.40 (3.23)	0.41, 2.37	2.81	0.006
	CR (69)	4.01 (2.64)			
MOCA	IMR (62)	5.32 (1.91)	0.86, 2.16	4.57	<0.001
	CR (69)	3.81 (1.87)			
HAMD	IMR (76)	−7.20 (3.08)	−3.03, −0.61	−2.98	0.003
	CR (77)	−5.38 (4.37)			
SDS	IMR (76)	−16.31 (9.30)	−8.67, −2.91	−3.97	<0.001
	CR (77)	−10.34 (8.73)			

**Table 4:** Summary of Adverse Reactions to Acupuncture and Herbal Medicine (N = 177).

Symptom (Test)	Mild		Moderate		Severe		No adverse (Normal)	
	N	%	N	%	N	%	N	%
Bleeding	101	57.06	–	–	–	–	76	42.94
Local hematoma	13	7.34	–	–	–	–	164	92.66
Unbearable prickling	14	7.91	–	–	–	–	163	92.09
Pallor	2	1.13	–	–	–	–	175	98.87
Sweating	1	0.56	–	–	–	–	176	99.44
Dizziness	2	1.13	–	–	–	–	175	98.87
Fainting	–	–	–	–	–	–	177	100
Allergies	–	–	–	–	–	–	177	100
Gastrointestinal discomfort	4	2.26	–	–	–	–	173	97.74
Blood RT (0 week)	23	12.99	–	–	–	–	154	87.01
Blood RT (8 week)	14	7.91	–	–	–	–	163	92.09
Urine RT (0 week)	35	19.77	–	–	–	–	142	80.23
Urine RT (8 week)	29	16.38	–	–	–	–	148	83.62
Stool RT (0 week)	–	–	–	–	–	–	177	100
Stool RT (8 week)	–	–	–	–	–	–	177	100
Kidney function test (0 week)	8	4.52	–	–	–	–	169	95.48
Kidney function test (8 week)	8	4.52	–	–	–	–	169	95.48
Liver function test (0 week)	39	22.03	–	–	–	–	138	77.97
Liver function test (8 week)	32	18.08	–	–	–	–	145	81.92

Mild adverse reactions: asymptomatic diagnostic finding, intervention not indicated; Moderate adverse reactions: Symptomatic, not interfering with ADL, and repair or revision not indicated; Severe adverse reactions: Symptomatic interfering with ADL; repair or revision indicated.

Bleeding in this table refers to local acupuncture site bleeding after the withdrawal of the needle, not internal bleeding, or serious forms of bleeding. The scalp is a site dense with capillaries, and scalp acupuncture can result in a small amount of bleeding from a drop of blood to several drops of blood at the local site, which stops quickly upon applying pressure for a few seconds to a few minutes.

**Table 5:** Prescriptions of Acupuncture and Chinese medication.

Function/Indication	Points/Formula	Location/Herbs
**Acupuncture**		
Basic prescription (Scalp acupuncture)	MS-6 Motor area (Dingnieqianxiexian)	0.5 cms posterior to the midpoint of the anterior-posterior line defines the upper limit of the motor area. The lower limit intersects the eyebrow-occiput line at the anterior border of the natural hairline on the temple.
	MS-7 Sensory area (Dingniehouxiexian)	A line parallel to the motor area and 1.5 cms behind it.
Basic prescription (Body acupuncture for the affected side of upper limb)	LI15 (Jianyu)	In the depression distal and anterior to the acromion, between the clavicular and acromial portions of the deltoid muscle.
	LI11 (Quchi)	With the elbow flexed, on the lateral end of the elbow crease, in a depression between the end of the crease and the lateral epicondyle of the humerus, on the extensor carpi radialis longus muscle.
	LI10 (Shousanli)	2 cun distal to LI11, on the extensor carpi radialis longus muscle.
	TE5 (Waiguan)	2 cun proximal to the dorsal wrist joint space (‘dorsal wrist crease’), between the radius and the ulna.
	LI4 (Hegu)	On the radial aspect of the hand, between the 1st and 2nd metacarpal bones, closer to the 2nd metacarpal bone and approximately at its midpoint.
Basic prescription (Body acupuncture for the affected side of lower limb.)	ST32 (Biguan)	Inferior to the anterior superior iliac spine and lateral to the sartorius muscle, at the level of the lower border of the pubic symphysis.
	ST36 (Zusanli)	3 cun distal to ST-35 (‘lateral eye of the knee’) and 1 fingerbreadth lateral to the anterior crest of the tibia, on the tibialis anterior muscle.
	GB34 (Yanglingquan)	In the depression anterior and inferior to the head of the fibula, between the peroneus longus and extensor digitorum longus muscles.
	GB39 (Xuanzhong)	3 cun proximal to the highest prominence of the lateral malleolus, on the anterior border of the fibula.
	BL60 (Kunlun)	In the depression on the line connecting the Achilles tendon and the highest prominence of the lateral malleolus.
For CI	GV20 (Baihui)	At the junction of a line connecting the apices of the ears and the midline, 5 cun from the anterior or 7 cun from the posterior hairline respectively.
	GV24 (Shenting)	On the midline, 0.5 cun superior to the anterior hairline.
	GB13 (Benshen)	On the midline, 0.5 cun superior to the anterior hairline).
	EX-HN1 (Sishencong)	A group of four points, each located 1 cun from GV20 (anterior, posterior and lateral).
	Temple-Three-Needles (Niesanzhen)	In the temple area, on the opposite side of the hemiplaegia, the first needle is located in 2 cun straight above ear apex, then the second and third needles are separately located at the lateral 1 cun of the first needle.
For PSD	LR3 (Taichong)	1st and 2nd metatarsal bones, in the depression proximal to the metatarsophalangeal joints and the proximal angle between the two bones.
	PC6 (Neiguan)	2 cun proximal to the anterior wrist joint space (‘most distal wrist crease’), between the tendons of the palmaris longus and flexor carpi radialis muscles.
	GV20 (Baihui)	At the junction of a line connecting the apices of the ears and the midline, 5 cun from the anterior or 7 cun from the posterior hairline respectively.
	Ex-HN-3 (Yintang)	On the anterior midline, between the eyebrows.
	GV24 (Shenting)	On the midline, 0.5 cun superior to the anterior hairline or 4.5 cun anterior to → Du-20.
For Type 1	LR2 (Xingjian)	Between the 1st and 2nd toes, proximal to the margin of the interdigital web.
	LR3 (Taichong)	On the dorsum of the foot, between the 1st and 2nd metatarsal bones, in the depression proximal to the metatarsophalangeal joints and the proximal angle between the two bones.
	LR14 (Qimen)	In the 6th intercostal space, on the mamillary line or 4 cun lateral to the midline.
For Type 2	SP10 (Xuehai)	With the knee flexed, 2 cun proximal and slightly medial to the medial superior border of the patella, in a depression on the vastus medialis muscle.
	ST40 (Fenglong)	At the midpoint of the line joining ST-35 and ST-41, 2 fingerbreadths lateral to the anterior crest of the tibia.
For Type 3	SP6 (Sanyinjiao)	3 cun proximal to the highest prominence of the medial malleolus, on the posterior border of the medial crest of the tibia.
	KI3 (Taixi)	In the depression between the highest prominence of the medial malleolus and the Achilles tendon.
	LR3 (Taichong)	On the dorsum of the foot, between the 1st and 2nd metatarsal bones, in the depression proximal to the metatarsophalangeal joints and the proximal angle between the two bones.
For Type 4	CV6 (Qihai)	On the anterior midline, 1.5 cun inferior to the umbilicus.
	CV4 (Guanyuan)	On the anterior midline, 3 cun inferior to the umbilicus.
	BL17 (Geshu)	1.5 cun lateral to the posterior midline, on the level of the lower border of the spinous process of the 7th thoracic vertebra (T7).
**Chinese herbal medicine**		
Type 1	Tian Ma Gou Teng decoction	Tian Ma 9 g, Gou Teng 15 g, Shi Jue Ming 15 g, Shan Zhi Zi 9 g, Huang Qin 9 g, Niu Xi 15 g, Du Zhong 12 g, Yi Mu Cao 15 g, Sang Ji Sheng 15 g,Ye JiaoTeng 9 g, Fu Sheng 9 g, raw Long Gu 30 g, raw Mu Li 30 g
Type 2	Ban Xia Bai Zhu Tian Ma decoction and Tao Hong Si Wu decoction	Ban Xia 9 g, Bai Zhu 9 g, Tian Ma 9 g, Fu Ling 9 g, Ju Hong 6 g, Sheng Di 15 g, Dang Gui 15 g, Chuan Xiong 9 g, Tao Ren 9 g, Hong Hua 6 g
Type 3	Zhen Gan Xi Feng decoction	raw Long Gu 15 g, raw Mu Li 15 g, Dai Zhe Shi 30 g, Gui Ban 15 g, Bai Shao 15 g, XuanShen 15 g, Tian Dong 15 g, ChuanLianZi 6 g, Yin Chen 6 g, Chuan Xiong 15 g, raw Mai Ya 6 g, fried Gan Cao 6 g
Type 4	Bu Yang Huan Wu decoction	raw Huang Qi 30 g, Dang Gui 15 g, Tao Ren 6 g, Hong Hua 6 g, Di Long 12 g, Chi Shao 15 g
For CI	–	Shi Chang Pu 15 g, Yi Zhi Ren 20 g, Yuan Zhi 9 g
For PSD	–	Chai Hu 9 g, Yu Jin 9 g, Bai He 9 g

Abbreviations: CI, cognitive impairment; PSD, post-stroke depression; Type 1, Disturbance of wind-fire; Type 2, Phlegm-stasis blocking collateral; Type 3, Yin deficiency and wind act; Type 4, Qi deficiency and blood stasis.

The standardized point name terminology comes from *Standard Acupuncture Nomenclature* (Second Edition. World Health Organization 1993). Scalp acupuncture points and the three-temple points are not included in the WHO materials, but are standardized by other conventions in the field; Point location terminology comes from the *WHO standard acupuncture point locations in the Western Pacific Region* (World Health Organization, Western Pacific Region, 2008) and the *Atlas of acupuncture* (Focks C. Elsevier Health Sciences, 2008). Chinese Pinyin, Latin, and Common Names of Chinese Herbs in the Study are detailed in Supplemental Table S3.
