# Supplementary material for: Comprehensive rehabilitation with integrative medicine for subacute stroke: A multicenter randomized controlled trial
Source: Sci Rep. 2016 May 13;6:25850. doi: 10.1038/srep25850 (PMC4865744; doi:10.1038/srep25850)
Supplement: Supplementary Information [file srep25850-s1.pdf]

Comprehensive rehabilitation with integrative medicine for subacute stroke: A multicenter randomized controlled trial

Jianqiao Fang Ph.D. M.D.[*1,2] fangjianqiao7532@163.com

Lifang Chen Ph.D. M.D.[1] clfang@163.com

Ruijie Ma Ph.D. M.D.[1] maria7878@sina.com

Crystal Lynn Keeler Ph.D.[2] Crystal.clk@gmail.com

Laihua Shen M.D.[3] slh86ly@163.com

Yehua Bao M.D.[4] byh@hz.cn

Shouyu Xu Ph.D. M.D.[5] overnightjo@msn.com

[1] Department of Acupuncture, The Third Affiliated Hospital of Zhejiang Chinese Medical University, 219 Moganshan Road, Xihu District, Hangzhou City, Zhejiang Province 310005, China

[2] Zhejiang Chinese Medical University, 548 Binwen Road, Binjiang District, Hangzhou City, Zhejiang Province 310053, China

[3] Department of Acupuncture & Encephalopathy, Jiaxing Hospital of Traditional Chinese Medicine. 1501 Zhong shan East Road, Jiaxing City, Zhejiang Province 310012, China

[4] Department of Acupuncture & Rehabilitation, Hangzhou Hospital of Traditional Chinese Medicine. 453 Tiyuchang Road, Xihu District, Hangzhou City, Zhejiang Province 310007, China

[5] Department of Rehabilitation, The Third Affiliated Hospital of Zhejiang Chinese Medical University, 219 Moganshan Road, Xihu District, Hangzhou City, Zhejiang Province 310005, China

[*]Corresponding. fangjianqiao7532@163.com

Supplemental Table S1. Variables at Different Stages, Mean (SD)

Variables	Group(n)	Week 0	Week 4	Week 8	Week 20
MBI	IMR(176)	39.11(15.56)	52.47(16.93)	66.04(17.09) **	75.36(16.64) **
	CR(172)	39.03(17.36)	49.58(18.30)	59.56(19.62)	66.64(19.60)
NIHSS	IMR(176)	10.06(3.88)	7.92(3.87)	5.63(3.39) **	3.69(2.85) **
	CR(172)	10.61(4.35)	8.76(4.54)	6.87(4.08)	5.67(3.82)
FMA	IMR(176)	43.86(16.07)	56.20(18.15)	69.01(17.75) **	77.44(16.84) **
	CR(172)	44.23(18.52)	52.66(19.80)	61.55(19.19)	68.68(18.03)
MMSE	IMR(62)	16.02(3.57)	16.66(3.63)	19.03(4.04)	21.42(4.47) *
	CR(69)	15.70(3.43)	16.16(3.25)	18.28(3.56)	19.71(3.69)
MoCA	IMR(62)	14.65(3.10)	15.11(3.06)	17.21(3.18)	19.97(3.00) **
	CR(69)	13.96(3.14)	14.46(2.92)	16.17(2.66)	17.77(2.74)
HAMD	IMR(76)	22.97(4.42)	21.74(4.45)	19.80(4.16)	15.78(4.09) *
	CR(77)	23.06(6.50)	22.57(6.33)	20.99(5.47)	17.69(5.32)
SDS	IMR(76)	64.93(5.40)	63.42(6.47)	57.77(7.87)	48.80(9.34) *
	CR(77)	63.95(6.32)	63.28(6.68)	59.60(6.78)	53.61(8.68)

* $P<0.05$, compared with CR group; ** $P<0.01$, compared with CR group.

Supplemental Table S2. Adverse Events Reported in Study

	IMR (n=180)	CR (n=180)
AEs	103	107
AE relatedness to treatment		
Not related	96	99
Possibly/definitely	2	2
Unknown	5	6
Study treatment		
Temporarily interrupted	2	3
Permanently discontinued	0	0
SAEs	13	11
SAE criteria		
Death	0	0
Life-threatening	0	0
Prolonged hospitalization	8	8
Resulted in disability	1	1
Important medical event	2	1
Other	2	1
SAE relatedness to treatment		
Not related	11	10
Possibly/definitely	1	0
Unknown	1	1
Study treatment		

Temporarily interrupted	1	2
Permanently discontinued	1	2
Five most common SAEs		
Pneumonia	6	5
Urinary tract infection	4	4
Heart failure	2	1
Acute heart disease	1	0
Recurrent stroke	0	1

Abbreviations: AE, adverse event; SAE, serious adverse event (FAS).

Supplemental Table S3. Chinese Pinyin, Latin, and Common Names of Chinese Herbs in the Study

Chinese Pinyin Names	Latin Names	Common Names
Tian Ma	Gastrodia elata Bl	Rhizoma Gastrodiae
Gou Teng	Uncaria rhynchophylla （Miq.）	Ramulus Uncariae Cum Uncis
Shi Jue Ming	Haliotis diversicolor Reeve	Concha Haliotidis
Shan Zhi Zi	Gardenia jasminoides Ellis	Fructus Gardeniae
Huang Qin	Scutellaria baicalensis Georgi	Radix Scutellariae
Niu Xi	Cyathula officinalis Kuan (Achyranthes bidentata Blume)	Radix Cyathulae (Radix Achyranthes)
Du Zhong	Eucommia ulmoides Oliv	Cortex Eucommiae
Yi Mu Cao	Leonurus heterophyllus Sweet	Herba Leonuri
Sang Ji Sheng	Taxillus chinensis (DC.) Danser	Herba Taxilli
Ye Jiao Teng	Caulis Polygoni Multiflori (Polygonum multiflorum Thunb.)	Tuber Fleeceflower Stem
Fu Shen	Poria cum Radix Pini	Poria with Hostwood
Long Gu	Fossilia Ossia Mastodi	Os Draconis
Mu Li	Ostrea gigas Thunberg	Concha Ostreae
Ban Xia	Pinellia ternata	Rhizoma Pinelliae
Bai Zhu	Atractylodes macrocephala Koidz	Rhizoma Atractylodis Macrocephalae
Fu Ling	Poria cocos (Schw.)Wolf	Poria
Ju Hong	Citrus grandis (L.) Osbeck (Citrus reticulate Blanco)	Exocarpium Citri Grandis (Exocarpium Citri Reticulatae rubrum)

Sheng Di	Rehmannia glutinosa Libosch	Radix Rehmanniae
Dang Gui	Angelica sinensis (Angelica sinensis (Oliv.) Diels)	Radix Angelicae Sinensis
Chuan Xiong	Ligusticum chuanxiong Hort.	Rhizoma Chuanxiong
Tao Ren	Prunus persica (L.) Batsch	Semen Persicae
Hong Hua	Carthamus tinctorius L.	Flos Carthami
Dai Zhe Shi	Haematitum	Haematitum
Gui Ban	Chinemys reevesii (Grey)	Carapax Et Plastrum Testudinis
Bai Shao	Paeonia lactiflora Pall.	Radix Paeoniae Alba
Xuan Shen	Scrophularia ningpoensis Hemsl.	Radix Scrophulariae
Tian Dong	Asparagus cochinchinensis （Lour.）Merr.	Radix Asparagi
Chuan Lian Zi	Melia toosendan Sieb. and Zucc.	Fructus Toosendan
Yin Chen	Artemisia capillaris Thunb.	Herba Artemisiae Scopariae
Mai Ya	Hordeum vulgare L.	Fructus Hordei Germinatus
Gan Cao	Glycyrrhiza uralensis Fisch.	Radix Glycyrrhizae
Huang Qi	Astragalus membranaceus (Fisch.)	Radix Astragali
Di Long	Pheretima aspergillum (E. Perrier)	Pheretima
Chi Shao	Paeonia lactiflora Pall.	Radix Paeoniae Rubra
Shi Chang Pu	Acorus tatarinowii Schott.	Rhizoma Acori Tatarinowii
Yi Zhi Ren	Alpinia oxyphylla Miq.	Fructus Alpiniae Oxyphyllae
Yuan Zhi	Polygala tenuifolia Willd.	Radix Polygalae
Chai Hu	Bupleurum chinense DC.	Radix Bupleuri

Yu Jin	Curcuma rcenyujin Y. H. Chenet C.Ling (Curcuma wenyujin Y. H. Chen and C. Ling)	Radix Curcumae
Bai He	Lilium brownii F.E. Brown var. viridulum Baker	Bulbus Lilii
